# Hepatic encephalopathy as a result of ammonia-induced increase in GABAergic tone with secondary reduced brain energy metabolism

**DOI:** 10.1007/s11011-024-01473-x

**Published:** 2024-11-19

**Authors:** Michael Sørensen, Jens Velde Andersen, Peter Nissen Bjerring, Hendrik Vilstrup

**Affiliations:** 1 Department of Internal Medicine, Viborg Regional Hospital, Viborg, Denmark; 2 Department of Hepatology & Gastroenterology, Aarhus University Hospital, Aarhus, Denmark; 3 Department of Drug Design and Pharmacology, Faculty of Health and Medical Sciences, University of Copenhagen, Copenhagen, Denmark; 4 Department of Intestinal Failure and Liver Diseases, Rigshospitalet, Copenhagen, Denmark

**Keywords:** Oxygen demand, CBF, Liver insufficiency, Organic delirium, Glutamine

## Abstract

Hepatic encephalopathy (HE) is a neuropsychiatric syndrome caused by liver insufficiency and/or portosystemic shunting. HE is mostly episodic and as such reversible. Hyperammonemia clearly plays a key role in the pathophysiology, but the precise detrimental events in the brain leading to HE remain equivocal. Several pathogenic models have been proposed, but few have been linked to clinical studies and observations. Decreased oxygen metabolism is observed in both type A and C HE and in this review, we advocate that this reflects an actual reduced oxygen demand and not a primary cause of HE. As driving force, we propose that the hyperammonemia via astrocytic glutamine synthetase causes an increased γ-aminobutyric acid (GABA) mediated neuro-inhibition which subsequently leads to an overall decreased energy demand of the brain, something that can be enhanced by concomitant neuroinflammation. This also explains the reversibility of the condition.

## Introduction

Hepatic encephalopathy (HE) is a severe but fluctuating and generally reversible neuropsychiatric syndrome caused by liver insufficiency and/or portosystemic shunting with symptoms ranging from subtle motoric and cognitive changes to coma (AASLD and EASL 2014). While it is generally accepted that hyperammonemia plays a key role in the pathogenesis of HE and that the HE diagnosis should be reevaluated in a liver patient with normal blood ammonia, the precise pathophysiological events in the brain remain equivocal. Decreased brain oxygen metabolism has been described in both episodic HE type C (cirrhosis) and type A (acute liver failure) and in this review, we analyze these findings in light of experimental studies. Despite the clinical dissimilarities, we include observations from both type A and C because they together contribute towards the mechanistic understanding of HE. Patients with non-cirrhotic portal hypertension such as portosystemic shunts without liver insufficiency are also in risk of developing HE (viz. type B) (Gioia et al. 2021) which is likely a combination of systemic hyperammonemia and gut-derived inflammation (see *Role of neuroinflammation* below), but these patients are sparsely investigated and thus not included here.

**Fig. 1:**
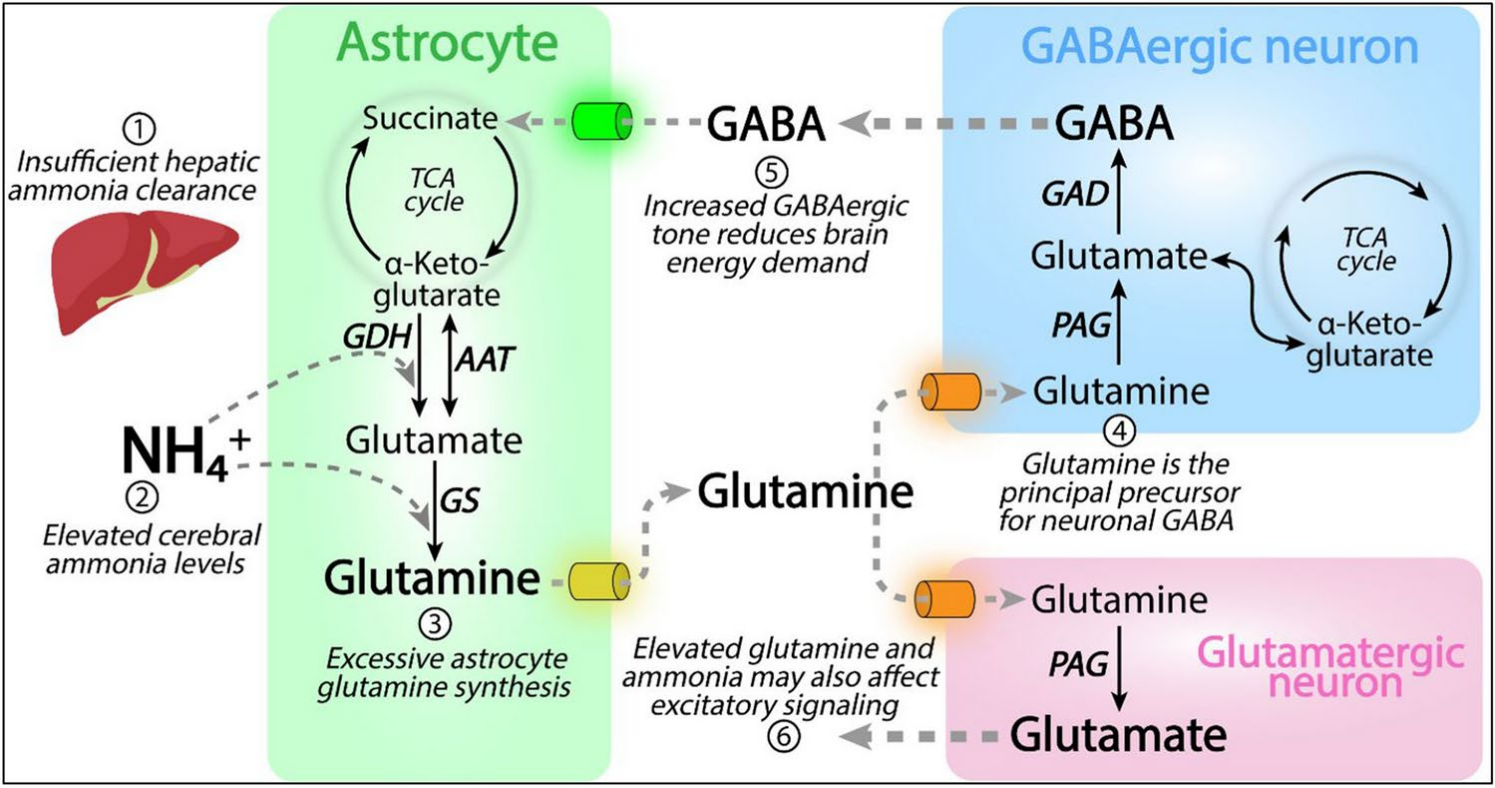
Proposed model for hyperammonemia-induced hepatic encephalopathy (HE) in liver insufficiency. Increased levels of circulating blood ammonia (NH_4_^+^) lead to increased flux into astrocytes where it is fixed in glutamine. Glutamine is used as precursor for γ-aminobutyric acid (GABA) in GABAergic neurons which subsequently leads to increased GABAergic tone with inhibition of neuronal activity and thus energy demand. Astrocytes also provide glutamine for glutamatergic (excitatory) neurons, but GABAergic neurons exert a powerful control over most of the excitatory signaling (see text for discussion). Abbreviation not explained above: AAT, aspartate aminotransferase; GDH, glutamate dehydrogenase GS, glutamine synthetase; PAG, phosphate-activated glutaminase; TCA cycle, tricarboxylic acid cycle

In this review, we focus on the observation that cerebral detoxification of increased blood ammonia into astrocytic glutamine via glutamine dehydrogenase (GDH) and glutamine synthetase (GS) leads to increased neuronal synthesis of the principal inhibitory neurotransmitter γ-aminobutyric acid (GABA) which leads to decreased neuronal activity. The lower neuronal activity in turn leads to decreased energy consumption with a concomitant drop in cerebral oxygen demand and blood flow (Fig. 1) (Sørensen et al. 2022).

## Cerebral oxygen metabolism and blood flow are reduced

Non-invasive positron emission tomography (PET) studies of awake patients with cirrhosis hospitalized with an episode of overt type C HE revealed lower cerebral metabolic rate of oxygen (CMRO_2_) and cerebral blood flow (CBF) in all brain regions including cerebellum when compared to patients without a history of HE and healthy controls. Once the patients recovered from HE, both CMRO_2_ and CBF increased to levels not different from the control groups (Iversen et al. 2009; Dam et al. 2013). These results are in line with a study from 1980 by Morgan et al. who, using arterial-venous blood measurements, showed that CMRO_2_, glucose metabolism, and CBF increased in patients with HE who were successfully treated with the dopamine antagonist bromocriptine (Morgan et al. 1980). In patients with type A HE, CMRO_2_ is also reduced (Aggarwal et al. 1994; Larsen et al. 1996a, b; Larsen et al. 1996a, b; Wendon et al. 1994; Strauss et al. 2003) though CBF reductions are more inconsistent due to the loss of cerebral autoregulation (discussed later). Overall, these clinical studies show that 1) reduced values of CMRO_2_ are related to the HE condition and not cirrhosis or liver failure in itself and that 2) in both episodic type C HE and type A HE, the reduction in CMRO_2_ is temporary and reversible. Next, we discuss how ammonia is the indirect cause of the decreased CMRO_2_.

## Cerebral uptake of blood ammonia and effect on cerebral energy metabolism

In all subjects (healthy controls, patients with cirrhosis with or without HE, and patients with acute liver failure) cerebral uptake of blood-borne ammonia increases linearly with increasing blood concentration of ammonia (Keiding et al. 2006; Strauss et al. 2001). Once inside astrocytes, ammonia is fixed in glutamine by GS (Fig. 1) (Norenberg and Martinez-Hernandez 1979). Astrocytes play a central physiological role in handling large amounts of ammonia by this formation of glutamine, which is released and taken up by neurons to replenish the GABA and glutamate neurotransmitter pools (Andersen and Schousboe 2023a, 2023b). High influx of ammonia from blood is thus likely to enter these pools, but the link to the pathophysiology of HE is still a matter of debate. It has been hypothesized that when astrocytes produce additional glutamine during hyperammonemia, it could lead to an increased flux of glutamine into the mitochondria where it is deamidated to glutamate and ammonia (known as the “Trojan horse hypothesis”). Through a series of events, ammonia should then be responsible for initiating the mitochondrial permeability transition (mPT) which leads to loss of mitochondrial function, astrocytic swelling and, ultimately, cell death (Albrecht and Norenberg 2006; Jayakumar et al. 2004; Rama Rao and Norenberg 2012). However, clinical data supporting this theory as relevant for development of HE are lacking and experimental studies of mitochondrial function in brain tissue exposed to increasing ammonia concentrations also do not support this as a pathophysiological explanation (Witt et al. 2017). Moreover, mPT is an irreversible process, something that does not fit with the clinical observations of readily normalization of CMRO_2_ after both type C (Dam et al. 2013; Morgan et al. 1980) and type A HE (Aggarwal et al. 1994). Another theory, which for a long time was considered a causal link between hyperammonemia and HE (Ott et al. 2005), was based on the observations that ammonia in isolated rat mitochondria inhibited the α-ketoglutarate dehydrogenase complex (α-KGDHC) which potentially could disrupt the tricarboxylic acid (TCA) cycle and thus deprive cells of energy (Lai and Cooper 1986), but in a series of cell and animal studies, we were unable to detect any such inhibition of ammonia on TCA cycle activity (Bak et al. 2009; Dadsetan et al. 2011; Johansen et al. 2007; Leke et al. 2011a, b; Leke et al. 2011a, b). On the contrary, ammonia actually increased TCA cycle activity in cultured mouse astrocytes and glutamatergic neurons (Johansen et al. 2007) and astrocytic and GABAergic co-cultures (Leke et al. 2011a, b). Interestingly, in bile-duct ligated rats, we found an increased shunt of ammonia via astrocytic TCA into neuronal GABA (Leke et al. 2011a, b). In GABAergic cell cultures, this detoxification of ammonia via GABA production increases oxidative metabolism (Leke et al. 2011a, b). Obviously, there would be no inhibitory effect of increased GABA production in cell cultures, but *in vivo*, these results fit with episodic hyperammonemia leading to increased production of GABA which decreases neuronal activity, as seen in patients with HE. Since restoration of ion gradients following excitatory signaling is a major energy expense in the brain, (Erecinska and Silver 1989) the theory of increased GABAergic tone leading to decreased oxygen metabolism is plausible and is in accordance with a human ^11^C-acetate PET-study in which we showed that during HE, it is the oxidative metabolism in neurons, not astrocytes, that is decreased (Iversen et al. 2014). That a supply limited oxidative metabolism is not a driving force of HE and that GABA is increased has also been validated by experimental findings by other groups (Mosso et al. 2022; Rackayova et al. 2016; Braissant et al. 2019; Cauli et al. 2009; Ferenci et al. 1983). Clinically, the effects of the competitive GABA-receptor antagonist flumazenil (Goh et al. 2017; Goulenok et al. 2002) and the newer GABA_A_ receptor-modulating steroid antagonist golexanolone on HE, are likewise in accordance with a causal role of GABA in the pathogenesis of HE (Johansson et al. 2015; Montagnese et al. 2021). Next, we discuss the relationship between decreased metabolic demand and the observed changes in CBF during HE.

## Cerebral blood flow and autoregulation

One of the keystones in maintaining a constant CBF over a range of arterial blood pressure is cerebral autoregulation and the decreased CBF observed during HE could thus hypothetically be explained by disturbed autoregulation. So far, clinical evidence of disrupted cerebral autoregulation in patients with chronic liver disease has been inconsistent, but it seems that most patients have preserved CBF autoregulation until late-stage, whereas it is disrupted in all patients with acute liver failure (Dethloff et al. 2008; Larsen et al. 1995a, b; Larsen et al. 1994; Larsen et al. 1995a, b; Strauss et al. 1998, 2000). Exposure of brain tissue to high concentrations of ammonia increases local CBF, probably via extracellular adenosine release (Bjerring et al. 2018a, b), but based on the clinical studies of CBF, this effect appears to be overruled by the metabolic effects of ammonia that decreases CBF during an episode of HE (Bjerring et al. 2018a, b; Dam et al. 2013; Iversen et al. 2009). This was also corroborated by an *in vivo* biosensor study in rats which showed that ammonia-induced increases in adenosine was not related to cerebral complications in ALF and thus likely overruled by other metabolic events in the brain (Bjerring et al. 2015). Analysis of CMRO_2_ in relation to CBF and oxygen levels in arterial blood from the Iversen et al. study also showed a maintained blood flow-metabolism coupling with no oxygen limitations due to decreased CBF or low arterial supply (Gjedde et al. 2010; Iversen et al. 2009). These observations are in line with observations in patients with acute liver failure exhibiting decreased CMRO_2_ and defunct autoregulation, but high internal jugular bulb saturation greater than 50% and a perfusion tightly matching the oxidative metabolism (Strauss et al. 2003). Experimental studies in rats support the clinical data since CBF autoregulation was only lost in models with a great loss of hepatocytes (Dethloff et al. 2008). Severe hypoxia has also been shown to induce a swelling of endothelial cells that impedes capillary blood flow, the so-called “no re-flow phenomenon” leading to irreversible hypoxic brain damage (Ames et al. 1968). Thus, the reversibility of CBF in patients recovering from HE speaks against hypoxia as a significant contributing factor and the decreased CBF observed during an episode of HE type C and in patients with type C HE is not due to lack of CBF autoregulation but is indeed secondary to a reduced metabolic demand which also explains why the changes are reversible.

## Cerebral lactate metabolism

Elevated brain lactate is a common observation in HE, yet the cause and cellular origin remains unclear (Rose 2010). Even under normal conditions are astrocytes net lactate producers (Barros et al. 2021) and several experimental studies have shown increased lactate production in astrocytes following hyperammonemia (Leke et al. 2011a, b; Bosoi and Rose 2014; Bosoi et al. 2014; Chatauret et al. 2001; Rose 2010; Zwingmann et al. 2003). This could indicate that during HE astrocytes and/or neurons switch to anaerobic metabolism, but such transition is not supported by experimental data. Astrocytes have a large capacity for oxidative metabolism (Barros 2022) which is maintained during episodic type C HE (Iversen et al. 2014) and acute liver failure (Strauss et al. 2003) as well as in experimental studies of rats with liver insufficiency induced by 90% hepatectomy (Witt et al. 2017). Lactate appears to be primarily metabolized in neurons (Qu et al. 2000) and the potential existence and role of a lactate shuttle from astrocytes to neurons is a matter of debate and is yet to be determined (Bak and Walls 2018; Barros et al. 2021; Barros and Weber 2018). Recent research even suggests that lactate has a neuroprotective effect in acute brain injury (Roumes et al. 2023; Cerina et al. 2024). If a primary neuronal consumption of lactate is assumed, it is plausible that the observed lactate accumulation during HE is mediated by a lower neuronal energy demand rather than by a switch to anaerobic metabolism. As argued above, this lower neuronal energy demand is mediated by an increase in the GABA-mediated inhibition. However, several other mechanisms may underlie the heightened lactate levels during HE (Rose 2010) and further metabolic studies are warranted to explore the physiological roles of brain lactate in HE and other brain disturbances.

## Ammonia and brain water content

Another pathophysiological phenomenon observed in clinical and experimental studies of HE that needs to be addressed is the association between hyperammonemia and an increase in brain water content and—in particular related to type A HE—also the risk of development of intracranial hypertension and cerebral incarceration (Clemmesen et al. 1999; Mardini et al. 2011; Wright et al. 2010). The water accumulation is believed to be a consequence of 1) increased brain osmolality, primarily driven by the ammonia-driven glutamine accumulation (Balata et al. 2003; Bjerring et al. 2008, 2012) that can reach levels saturating the capacity of the amino transport proteins out of the brain in the blood–brain barrier (O'Kane and Hawkins 2003) and 2) alterations in the regulation of water and electrolyte fluxes, i.e., abnormal function of the so-called glymphatic system (Hadjihambi et al. 2019) and regional changes in expression of membrane channels such as aquaporin-4 (Eefsen et al. 2010), the Na^+^-K^+^-2Cl^−^ cotransporter isoform 1 (NKCC1) (Rangroo Thrane et al. 2013) and inwardly rectifying potassium channel (Kir4.1) (Elsherbini et al. 2022; Obara-Michlewska et al. 2011). The brain tissue water accumulation is in absolute numbers estimated to be at most a few percent or less (Eefsen et al. 2010; Larsen et al. 2013; Wright et al. 2010; Winterdahl et al. 2019) and is in itself not responsible for the clinical signs of brain dysfunction or the observed reduction in metabolic rates of oxygen. However, in the most severe cases of type A HE with poor survival, the oedema acts in concert with an impaired autoregulation of cerebral blood flow and unstable systemic hemodynamics which may lead to reduced cortical perfusion and irreversible brain damage. As such, the correlation between hyperammonemia and brain water content observed in ALF is probably due to the multi-organ loss of homeostasis in this condition and likely is why the same complication is not seen in type C HE.

## Role of neuroinflammation

While the main topic of the present review is effects of ammonia on brain energy metabolism via increased GABAergic tone, the role of neuroinflammation must also be addressed. As reviewed in detail elsewhere, patients with chronic liver disease show systemic inflammation which can induce neuroinflammation, i.e. activation of microglia and astrocytes which compose the innate immune cells of the brain (Cabrera-Pastor et al. 2019; Giuli et al. 2023). Chronic neuroinflammation leads to neurodegeneration with brain dysfunctions including cognitive impairment, depression, anxiety, and memory loss, all symptoms observed in HE (Bachiller et al. 2018). Hyperammonemia and circulation bile acids can also activate microglia and systemic inflammation enhances the harmful effects of ammonia on psychometric tests (Giuli et al. 2023; Shawcross et al. 2004). In addition, the gut-liver-brain axis also contributes to systemic inflammation, in part due to altered gut microbiota, but also due to liver insufficiency including portosystemic shunting (Giuli et al. 2023). Evidence of neuroinflammation in patients with chronic liver disease is however limited to a few post-mortem studies (Balzano et al. 2018; Dennis et al. 2014; Zemtsova et al. 2011). In hyperammonemic rats, activation of astrocytes and microglia initiates neuroinflammation cascades in cerebellum and alters expression of glutamate decarboxylase (GAD), GAT3 and GABA_A_-receptors which have been proposed as mechanisms leading to elevated cerebellar GABAergic tone in HE causing impaired motor coordination (Cabrera-Pastor et al. 2019; Hernandez-Rabaza et al. 2016; Llansola et al. 2024; Arenas et al. 2022). It must be noted though that cerebellar astrocytes display unique functions in relation to GABA homeostasis, including GABA synthesis via alternative pathways and subsequent GABA release through multiple proposed mechanisms (Andersen et al. 2023). Hence, whether neuroinflammation contributes to increased GABAergic tone in other brain regions besides the cerebellum during HE remains to be established.

## Discussion and concluding remarks

Pathophysiological studies of HE based on clinical observations and measurements are surprisingly sparse. However, there is no doubt that during HE, cerebral oxygen metabolism is reduced, and this reduction is reversible. The available clinical data, corroborated rather than refuted by experimental studies, further indicate that this is not caused by disrupted energy metabolism but reflects a true reduced metabolic demand resulting from astrocytic overproduction of glutamine which leads to an increased GABAergic tone that reduces neuronal energy consumption. CBF decreases because of decreased metabolic demand with intact blood flow-metabolism coupling. The idea of cerebral energy consumption being limited by compromised intracellular energy metabolism or macro- or micro perfusion as primary mechanism for causing HE is accordingly not supported by data from experiments focusing on brain cell and neurotransmitter metabolism.

Astrocytic glutamine production serves a crucial role in the neuronal replenishment of both glutamate and GABA (Andersen and Schousboe 2023b). It is estimated that approximately 90% of all synapses in the cerebral cortex are glutamatergic (Braitenberg et al. 1998) which means that a minority of GABAergic neurons exerts a powerful control over most of the excitatory signaling. An upregulation of astrocyte glutamine synthesis and release, as seen in HE, thus has functional effects on the signaling properties of the powerful GABAergic neurons. Pharmacological inhibition of GS depleted the total intracellular levels of GABA more prominently than glutamate in acutely isolated brain slices (Andersen et al. 2017) and impaired neuronal release of GABA more than that of glutamate when measured by *in vivo* microdialysis (Paulsen and Fonnum 1989). These observations suggest that astrocytic glutamine via GS is more important for GABA synthesis compared to glutamate synthesis. Yet, it remains unknown how sustained elevations in brain glutamine levels may differently affect GABAergic and glutamatergic transmission in vivo.

When comparing experimental studies with clinical studies, it emerges as a plausible mechanism of the decreased brain oxygen consumption in HE that hyperammonemia in the presence of liver disease leads to an increased shunting of ammonia into neuronal GABA. This in turn increases the inhibitory tone with decreased neuronal energy metabolism as a result. Besides providing a credible explanation for the decreased oxygen metabolism and cerebral blood flow observed in patients during HE, this also fits with the clinical presentation of the patients including the reversibility of the condition. It would expand the understanding of the condition to compare regional GABAergic tone with measurements of local oxygen metabolism and blood flow, something that hitherto has not been done.

Neuroinflammation undoubtedly also plays a role in HE, both directly and by amplifying the effects of hyperammonemia. The fluctuating nature of HE agrees with fluctuating ammonia levels in blood whereas long standing hyperammonemia and neuroinflammation together could result in irreversible neurological deterioration (Giuli et al. 2023; Llansola et al. 2024).

## Data Availability

No datasets were generated or analysed during the current study.
